# Phosphatidylinositol-4,5-Biphosphate (PI(4,5)P_2_) Is Required for Rapid Endocytosis in Chromaffin Cells

**DOI:** 10.1155/2020/9692503

**Published:** 2020-09-09

**Authors:** Fouad Azizi

**Affiliations:** Translational Research Institute, Academic Health System, Hamad Medical Corporation, Doha, Qatar

## Abstract

**Objective:**

Phosphoinositides play a regulatory role in clathrin-mediated endocytosis. However, their involvement in clathrin-independent endocytosis termed rapid endocytosis (RE), which is the mode of vesicle recycling during neurotransmitter release by transient fusion (known as kiss-and-run), has not been investigated. Here, we used patch-clamp recording of whole-cell membrane capacitance in adrenal chromaffin cells (ACC) to monitor changes of RE kinetics in response to pharmacological alteration of phosphatidylinositol-4,5-biphosphate (PI(4,5)P_2_) level by phenylarsine oxide (PAO) or antibody against phosphatidylinositol 4-kinase (Ab_PI4K_).

**Results:**

We found that PAO and Ab_PI4K_ significantly abrogated RE kinetics. Infusion of PI(4,5)P_2_ through the patch pipette potentiated RE kinetics and reversed PAO- and Ab_PI4K_-induced blockade of RE. Similarly, the application of the bifunctional thiol dithiothreitol (DTT) to PAO-treated cells completely prevented the inhibitory effect of PAO on RE. These findings indicate that PI(4,5)P_2_ is implicated in the signaling (mechanistic) process of RE in ACC.

## 1. Introduction

Phosphoinositides play a critical role in the nervous system functions, and their levels are normally tightly regulated by several classes of phosphoinositide kinases and phosphatases. The alteration of cellular pools of these lipids has been linked to a broad spectrum of neurological disorders (e.g., stroke, schizophrenia, bipolar disorder, and Alzheimer's disease) [1]. Especially, phosphoinositides are known to participate extensively in vectorial membrane traffic to and from the plasma membrane mainly through clathrin-dependent endocytosis [2–4], yet their role in clathrin-independent RE has not yet being investigated.

PI(4,5)P_2_ is the most abundant phosphoinositide at the inner surface of the plasma membrane where it interacts with cargo proteins leading to formation, scission, and uncoating of clathrin-coated vesicles (CCVs) [4–9] under the control of phosphoinositide kinases and phosphatases, which tightly regulate the spatio-temporal synthesis and turnover of PI(4,5)P_2_ [4, 8–10]. In fact, PI(4,5)P_2_ synthesis involves phosphorylation of phosphatidylinositol (PI) by several classes of PI 4-kinase (PI4K) leading to the formation of the intermediary precursor phosphatidylinositol 4-phosphate (PI4P), which undergoes phosphorylation by phosphatidylinositol-4-phosphate 5-kinase type I (PI5K) to generate PI(4,5)P_2_ [3, 4, 8, 9].

RE is the clathrin-independent fast vesicle recycling process that is associated with the transient fusion (known as “kiss-and-run”) mode of neurotransmission in ACC [11–13]. RE is a highly regulated process that involves the GTPase dynamin-1 [11, 13], which is a PI(4,5)P_2_-binding protein [5]. Importantly, PI(4,5)P_2_ binds and promotes the insertion of dynamin into the plasma membrane, thereby facilitating the scission of CCVs from the plasma membrane [4, 6, 8, 9]. Hence, we hypothesized that PI(4,5)P_2_ shall be required for RE.

In the present study, we set out to investigate the effect of PAO and anti-PI4K antibody on Ca^2+^-evoked exocytosis coupled to rapid endocytosis in ACC using patch-clamp recording of whole-cell membrane capacitance (Cm) under physiological conditions.

## 2. Materials and Methods

Phenylarsine oxide (P3075) and DL-dithiothreitol (D0632) were purchased from Sigma-Aldrich (Saint-Louis, MO, USA). The synthetic and water-soluble short-saturated fatty acid chain dioctanoyl phosphoinositide diC8-PI(4,5)P_2_ was obtained from Echelon Biosciences (P-4508, Salt Lake City, UT). The anti-phosphatidylinositol 4 kinase III alpha antibody (Ab_PI4K_) was from Abcam (ab111565, Cambridge, MA). Stock solutions of PAO (dissolved in DMSO at 10 mM) and DTT (dissolved in Tyrode' solution at 50 mM) were made fresh on the day of experiments, kept in ice, and from which working solutions were made in Tyrode' solution.

ACC were isolated by collagenase digestion of calf adrenal medullae and cultured as described previously [11, 12].

Whole-cell membrane capacitance (Cm) measurements in the standard whole-cell configuration were performed using an EPC-10 amplifier (HEKA Elektronik) as described previously [12, 14]. Briefly, Cm was evoked by a 50 mV (root mean square) sine wave at 1500 Hz using the manufacturer's Pulse software (HEKA Elektronik). Exocytosis coupled to RE was evoked by a brief stimulation protocol that consists in applying a pulse train of 10 voltage depolarizations [8, 10]. The patch pipette contained (in mM) 100 K-glutamate, 12 NaCl, 30 HEPES, 5 MgCl_2_, 2 ATP, 0.35 GTP, and 0.1 EGTA, pH adjusted to 7.2 with KOH. Ca^2+^ currents were recorded and quantitated as described [14]. The bath solution (Tyrode' solution) consisted of (in mM) 2 CaCl_2_, 10 HEPES, 10 glucose pH 7.2; 150 mM tetraethylammonium chloride and 1 *μ*M tetrodotoxin. All experiments were performed on cell culture dishes submitted to different treatments and were carried out at room temperature (≈25°C). Parallel control dishes (untreated cells) were used on the same day.

### 2.1. PAO and DTT Treatment

ACC were treated with PAO (5 *μ*M) for 5 min at 37°C in the CO_2_ incubator, then 0.5 mM DTT was added to the bath solution prior to whole-cell patch-clamp capacitance recording.

### 2.2. Ab_PI4K_ Treatment

The antibody, dialyzed against the internal pipette solution, was loaded into the patch pipette to a final concentration of 1 mg/ml prior to whole-cell patch-clamp capacitance recording. As a control, the antibody was heat inactivated at 70°C for 20 minutes.

### 2.3. PI(4,5)P_2_ Treatments

The phosphoinositide diC8-PI(4,5)P_2_ was dissolved in the patch pipette-filling internal solution, then dialyzed against the same solution to make 1 mM stock solution stored as aliquots at −80°C. The lipid solution was sonicated for 15 min on ice prior to loading into the patch pipette at a final concentration of 100 *μ*M.

### 2.4. Data Analysis

The raw data were pulled and computed from all the records. Data are expressed as the mean ± SEM (standard error of the mean). Statistical analysis was performed by 2-sided Student's *t*-test. Differences between experimental conditions resulting in *P* < 0.05 were considered statistically significant.

## 3. Results

### 3.1. PAO and Ab_PI4K_ Blocked Rapid Endocytosis

**Figure 1:**
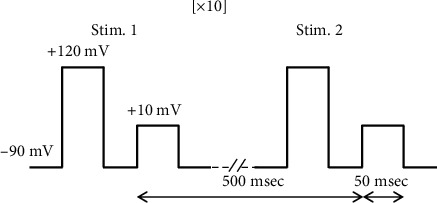
Stimulation protocol consisting of a train of 10 depolarizations, from a holding potential of −80 mV to +10 mV, each lasting 50 ms; 500 ms separated each depolarization; Each depolarization was preceded by a 50 ms prepulse to +120 mV to recruit facilitation Ca^2+^ channels [7, 8].

**Figure 2:**
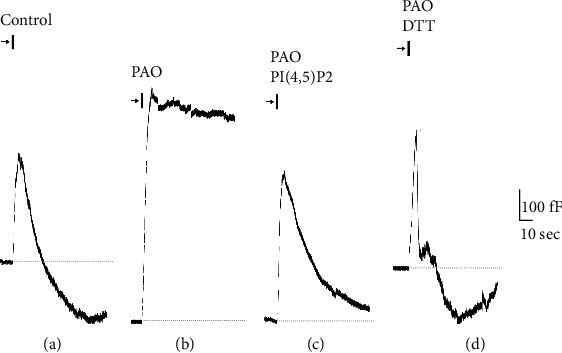
Inhibition of RE by PAO in chromaffin cells subjected to one round of stimulation. Representative whole-cell membrane capacitance (Cm) recordings from cells treated with (a) no drug (control), normal RE is seen following the exocytotic burst; (b) PAO (5 *μ*M); (c) PAO + PI(4,5)P_2_ (100 *μ*M); (d) PAO + DTT (0.5 mM). Arrows and bars above traces indicate the timing at which the stimulation protocol was executed to elicit secretion, and the dashed lines are baselines.

**Figure 3:**
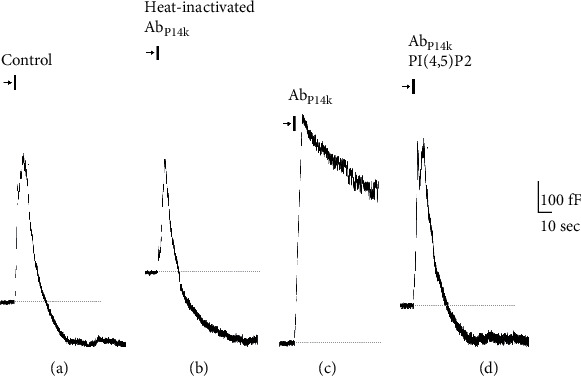
Inhibition of RE by Ab_PI4K_ in chromaffin cells subjected to one round of stimulation. The following representative capacitance (Cm) traces correspond to (a) untreated cells (control); (b) Heat-inactivated Ab_PI4K_ (1 mg/ml); (c) Ab_PI4K_ (1 mg/ml); (d) Ab_PI4K_ + PI(4,5)P_2_. Arrows and bars above traces indicate the timing at which the stimulation protocol was executed to elicit secretion, and the dashed lines are baselines.

In an attempt to characterize the effect of PAO and Ab_PI4K_ on RE, ACC was subjected to one round of stimulation (Figure 1). The readouts of Cm changes are illustrated in Figures 1 and 2. In untreated (control) cells, depolarization-activated Ca^2+^ influx triggered a biphasic Cm response. During the first phase, the stimulation caused stepwise increases in Cm that correspond to the fusion of dense-core vesicles (DCVs) with the plasma membrane and release of catecholamines (exocytosis) (Figures 2(a) and 3(a)). This phase is immediately followed by a rapid decline in Cm due to recapture of the membrane by RE, which is completed within ≈35 sec as previously documented [11]. However, the arsenical compound PAO completely abolished RE (Figure 2(b)), resulting in unrecovered DCVs and a subsequent boost of exocytosis by 2-fold (±0.06) without affecting Ca^2+^ current (Table 1). Similarly, Ab_PI4K_ inhibited RE though not efficiently as PAO (Figure 3(c)) and consequently increased membrane capacitance (i.e., exocytosis) by 1.6-fold (±0.05) when compared to control (Table 1). Note that heat-inactivated Ab_PI4K_ did not affect RE kinetics demonstrating the specificity of the antibody (Figure 3(b)).

### 3.2. PI(4,5)P_2_ Rescued Inhibition of Rapid Endocytosis by PAO and Ab_PI4K_

In ACC, the inhibition of PI4K enzymatic function by Ab_PI4K_ or PAO is expected to substantially reduce the endogenous level of PI(4)P, which is needed for replenishment of plasma membrane PI(4,5)P_2_ [3, 15]. Hence, we reasoned that the introduction of exogenous PI(4,5)P_2_ into ACC via the patch pipette would be a good strategy to counteract the inhibitory action of PAO and Ab_PI4K_ on RE. Notably, infusing PI(4,5)P_2_ into untreated patched ACC submitted to one round of stimulation enhanced RE by 2-fold (±0.04) in comparison to control (Table 1), indicating that fine-tuning of cellular PI(4,5)P_2_ level (turnover) regulates vesicle recycling and replenishment rate of the secretory pools. Repeating such maneuver in ACC treated with PAO resulted in membrane (vesicle) retrieval albeit with slower kinetics compared to control (Figure 1(c), Table 1). In contrast, exogenous PI(4,5)P_2_ completely rescued RE in cells treated with Ab_PI4K_ (Figure 2(d)), indicating that PI(4,5)P_2_ is required for RE. Evoked secretion and Ca^2+^ current remained intact mirroring control conditions (Table 1).

### 3.3. DTT Prevented Inhibition of Rapid Endocytosis by PAO

The mechanism by which PAO reacts with biological molecules and inhibits certain enzymatic processes involves crosslinking their vicinal sulfhydryl groups with the arsenic atom to form a stable ring complex that can be reversed with a stoichiometric amount of a vicinal dithiol compound, such as DTT [16]. Indeed, the addition of DTT in the bath solution averted the inhibitory effect of PAO on RE (Figure 1(d)). Cm measurements revealed that secretion and Ca^2+^ current were not affected by DTT, and the kinetics of RE were very similar to control conditions (Table 1).

## 4. Discussion

To our knowledge, this is the first whole-cell membrane capacitance (Cm) patch-clamp electrophysiological study to use PAO and Ab_PI4K_ as pharmacological blockers in order to investigate the potential role of phosphoinositides in RE. In ACC, one round of exocytosis coupled to rapid endocytosis was evoked by a physiological stimulation protocol that has been designed specifically for recruiting facilitation L-type Ca^2+^ channels, which are so closely linked to secretory sites [17, 18]. Blocking these channels would of course block the whole process of secretion but none of the drugs affected the Ca^2+^ current (cumulative current integral), which was very similar in the different experimental conditions (Table 1). Interestingly, PAO at 100 *μ*M was found to transiently stimulate basal L-type Ca^2+^ current in cardiomyocytes [19]. In contrast, at motor nerve endings of neuromuscular junctions, PAO at 30 *μ*M was shown to inhibit Ca^2+^ entry via N-type Ca^2+^ channels [20], but this type of channels contributes little to evoked secretion in chromaffin cells [17, 18].

PAO, a sulfhydryl (SH)-reactive agent, has been widely used to investigate the role of PI(4,5)P_2_ in intracellular anterograde and retrograde vesicular transport as this trivalent arsenical chemical blocks PI4K activity and thereby reduces PI4P and PI(4,5)P_2_ levels [3, 15, 16, 21]. In particular, PAO was shown to block clathrin-dependent endocytosis through the depletion of plasma membrane PI(4,5)P_2_ [22]. In chromaffin cells [15] and isolated synaptosomes [21], PAO was found to reduce catecholamine secretion through the inactivation of PI4K and concomitant loss of PI(4)P and PI(4,5)P_2_ pools. In this study, we show that PAO exhibited an inhibitory effect on the rapid clathrin-independent endocytosis without affecting exocytosis.

At low micromolar concentrations, PAO inhibits the type-III*α* isoform of PI 4-kinase (PI4K230), which is considered the main lipid-kinase responsible for the generation of plasma membrane phosphoinositides [16]. Furthermore, the effect of PAO treatment on plasma membrane PI4P and PI(4,5)P_2_ has been demonstrated using the biosensors of these lipids [3, 23, 24].

The complete abolition of RE induced by PAO but not by the anti-PI4K type III-*α* (PI4K230) antibody is likely due to the ability of PAO to target other PI4K isoforms [15, 16, 24]. For example, PAO was found to target the chromaffin granule-associated PI4K55 (PI4K type II-*α*) [15, 24]) resulting in the attenuation of Ca^2+^-stimulated neurotransmitter release due to reduced levels of both phosphoinositides PI4P and PI(4,5)P_2_ in permeabilized ACC [15] and isolated synaptosomes [21, 25]. Therefore, the residual endocytosis observed with Ab_PI4K_ may be due to the activity of other PI4K isoforms. A deeper investigation with specific inhibitors of PI4k isoforms would confirm the results found with PAO and Ab_PI4K_ and identify which PI4k isoforms are implicated in RE.

Loading ACC with exogenous PI(4,5)P_2_ superseded Ab_PI4K_- and PAO-evoked inhibition of RE. Similarly, the disulfide-reducing chemical agent DTT, which was found to reinstate endogenous PI4P and PI(4,5)P_2_ to their normal levels in PAO-treated ACC [15], completely restored RE. Therefore, PI(4,5)P_2_ is required for the fast recycling of DCVs and replenishing of the secretory pools for subsequent rounds of secretion.

## 5. Conclusions

In conclusion, we used two functional assays to provide proof of concept that PI(4,5)P_2_ is involved in the signaling of clathrin-independent rapid endocytosis associated with transient fusion of vesicles, which is a neurotransmission mode manifested in hippocampal neurons undergoing bursting patterns of activity implicated in tasks and activities such as memory formation [26]. We propose that PI(4,5)P_2_ may function in RE by facilitating assembly of dynamin-1 molecules [8, 9, 27] after their insertion into the plasma membrane [4–6, 8, 9]. The interaction of PI(4,5)P_2_ with the Pleckstrin homology domain (PH domain) of dynamin-1 would result in adequate stimulation of its intrinsic GTPase activity [8, 9, 27, 28] and consequently lead to efficient pinching off of endocytic vesicles. Evidence for such scenario has been already shown in pancreatic *β* cells where PI(4,5)P_2_ was found to promote DCVs kiss-and-run mode of insulin secretion [29]. Nevertheless, phosphatidylinositol-3,4-bisphosphate (PI(3,4)P_2_) is another important signaling lipid that is mechanistically involved in both clathrin-dependent endocytosis [6, 8, 30, 31] and clathrin-independent fast endophilin-mediated endocytosis (FEME) [31]. Indirect conversion of PI(4,5)P_2_ to PI(3,4)P_2_ is required during the late stage of the endocytic process in order to recruit PI(3,4)P_2_ effectors at endocytic sites and trigger vesicle scission and recycling by dynamin [6, 8, 30]. Therefore, PI(3,4)P_2_ and its effectors merit attention and investigation in RE. Future studies using double patch-clamp electrophysiology, which is based on combining whole-cell and cell-attached configurations [12], would reinforce our results and certainly shed more light on whether these phosphoinositides modulate neurotransmitter release by regulating fusion pore dynamics during individual secretory events in ACC. Additional work analyzing RE in cells transfected with mutants of the phosphatidylinositol 4-kinase (PI4K) isoforms would strengthen the findings of this study and identify the major PI4K involved in RE.

## Figures and Tables

**Table 1:** Statistical analysis of rapid endocytosis parameters in chromaffin cells.

Treatment	Cm increase (fF)	RE duration (sec)	Peak current (pA)	Cumulative current integral (pC)
Control (*n* = 22)	489.5 ± 15.8	30.1 ± 0.6	−599.2 ± 26.8	167.8 ± 7.5
PAO (*n* = 23)	962.1 ± 28.7^∗^	—	−558.3 ± 17.4	156.3 ± 4.9
Ab_PI4K_ (*n* = 21)	794.1 ± 27.3^∗^	—	−587.5 ± 30.6	164.5 ± 8.6
PI(4,5)P_2_ (*n* = 17)	488.2 ± 17.2	15.0 ± 0.8^∗∗^	−524.5 ± 24.8	146.9 ± 7.0
PAO + PI(4,5)P_2_ (n =19)	487.0 ± 14.6	64.1 ± 3.1^∗^	−538.9 ± 16.7	150.9 ± 4.7
Ab_PI4K_ + PI(4,5)P_2_ (*n* = 19)	440.6 ± 9.3	36.1 ± 2.5	−544.0 ± 19.5	152.3 ± 5.5
PAO + DTT (*n* = 15)	467.0 ± 8.5	29.7 ± 1.4	−507.0 ± 15.0	142.0 ± 2.9

Whole-cell membrane capacitance (Cm) records of exocytosis and rapid endocytosis (RE) were acquired and analyzed according to our previously described methods [7, 8]. Total Cm increase and total Cm decrease correspond respectively to the maximum increase and the maximum decrease of Cm induced by voltage depolarization (see Materials & Methods). The duration of RE is the time required for Cm to return to baseline from the maximum level after stimulation (^∗^*P* < 0.01, ^∗∗^*P* < 0.001, significantly different from control). Peak current corresponds to the maximum Ca^2+^ current amplitude evoked by the first pulse in the train of stimulation. Cumulative current integral is calculated from the total number of Ca^2+^ ions entering the cell during the entire stimulation period (10 depolarizations of 50 ms).

## Data Availability

All data generated or analyzed during this study are included in this published article.

## Abbreviations

PI4P: Phosphatidylinositol 4-phosphate
PI(4,5)P_2_: Phosphatidylinositol-4,5-biphosphate
PI(3,4)P_2_: Phosphatidylinositol-3,4-bisphosphate
RE: Rapid endocytosis
Cm: Whole-cell membrane capacitance
ACC: Adrenal chromaffin cells
PAO: Phenylarsine oxide
DTT: Dithiothreitol
PI: Phosphatidylinositol
PI4K: Phosphatidylinositol 4-kinase
PI5K: Phosphatidylinositol-4-phosphate 5-kinase
CCVs: Clathrin-coated vesicles
DCVs: Dense core vesicles
Ab_PI4K_: Antibody against phosphatidylinositol 4-kinase.
